# An Investigation of Ovarian and Adrenal Hormone Activity in Post-Ovulatory Cheetahs (*Acinonyx jubatus*)

**DOI:** 10.3390/ani12070809

**Published:** 2022-03-22

**Authors:** Diana C. Koester, Morgan A. Maly, Sarah Putman, Katie L. Edwards, Karen Meeks, Adrienne E. Crosier

**Affiliations:** 1 Conservation and Science Department, Cleveland Metroparks Zoo, Cleveland, OH 44109, USA; 2 Department of Biology, Case Western Reserve University, Cleveland, OH 44106, USA; 3 Smithsonian National Zoological Park and Conservation Biology Institute, Front Royal, VA 22630, USA; malym@si.edu or; 4 Department of Biological Sciences, North Carolina State University, Raleigh, NC 27695, USA; 5 Department of Molecular Biomedical Sciences, North Carolina State University, Raleigh, NC 27695, USA; 6 Smithsonian/Mason School of Conservation, Front Royal, VA 22630, USA; 7 North of England Zoological Society, Chester Zoo, Chester CH2 1LH, UK; k.edwards@chesterzoo.org; 8 White Oak Conservation, Yulee, FL 32097, USA; kmeeks@white-oak.org

**Keywords:** cheetah, hormone, pregnancy, glucocorticoid, progestagen, estrogen, artificial insemination

## Abstract

**Simple Summary:**

More than three decades of scientific study have been unable to determine the etiology of reproductive issues in cheetahs under human care. The reproduction of cheetahs in zoological facilities has never been self-sustaining, and the differences between females who establish pregnancy and those that do not remain poorly understood. The objective of this study was to examine and compare ovarian and adrenal hormones post-ovulation in pregnant and non-pregnant animals to better understand female physiology after natural breeding or artificial insemination, and determine what may be contributing to the frequent lack of success. The authors also sought to validate a urinary progestagen assay to assist with pregnancy detection. Although fecal glucocorticoid metabolites among pregnant and non-pregnant groups were not different, samples from the third trimester in pregnant animals were higher than at any other time. Additionally, glucocorticoids were higher, and estrogens tended to be lower in samples from pregnant females that gave birth to singletons, than those that had multi-cub litters. As a supplementary benefit, this is the first time urinary progestagens have been measured and have been able to distinguish pregnant and non-pregnant cheetahs. The results provide first-time insights into ovarian and adrenal hormonal events surrounding ovulation and pregnancy detection in cheetahs.

**Abstract:**

Cheetahs have been the subject of reproductive study for over 35 years, yet steroid hormone activity remains poorly described after ovulation. Our objective was to examine and compare fecal progestagen (fPM), estrogen (fEM), and glucocorticoid (fGM) metabolite concentrations post-ovulation in pregnant and non-pregnant animals to better understand female physiology (1) during successful pregnancy, (2) surrounding frequent non-pregnant luteal phases, and (3) after artificial insemination (AI) to improve the low success rate. Secondarily, the authors also validated a urinary progestagen metabolite assay, allowing pregnancy detection with minimal sample collection. Fecal samples were collected from 12 females for ≥2 weeks prior to breeding/hormone injection (the PRE period) through 92 days post-breeding/injection. Samples were assessed for hormone concentrations using established enzyme immunoassays. Urine samples were collected for 13 weeks from 6 females after natural breeding or AI. There were no differences among groups in fGM, but in pregnant females, concentrations were higher (*p* < 0.01) in the last trimester than any other time. For pregnant females that gave birth to singletons, fGM was higher (*p* = 0.0205), but fEM tended to be lower (*p* = 0.0626) than those with multi-cub litters. Our results provide insight into the physiological events surrounding natural and artificially stimulated luteal activity in the cheetah.

## 1. Introduction

Surviving in highly fragmented populations primarily in eastern and southern Africa, the wild cheetah (*Acinonyx jubatus*) is currently classified as vulnerable by the IUCN, with approximately 7100 individuals remaining [1]. To avoid larger predators like lions and hyenas, the majority of free-ranging cheetahs live on unprotected lands, where human conflict with livestock and game farmers continues to cause population declines and threatens the longevity of this species in the wild [1]. Steadily falling numbers of free-ranging cheetahs highlight the importance of maintaining a healthy and sustainable population of cheetahs in human care. Ex situ populations serve to educate and motivate the public to contribute to conservation efforts, as an insurance population to avoid extinction and provide individuals for reintroduction efforts, and finally, as the focus of research to better understand this highly unique species with work that would be impossible to conduct on wild counterparts. Cheetahs housed in North American facilities are part of a singularly managed group called the Species Survival Plan (SSP) and are considered as one cohesive research population containing approximately 358 animals at 52 institutions [2].

Over almost the last four decades, research utilizing the SSP population has yielded a substantial body of work, advancing our understanding of topics, such as species’ nutritional needs [3,4], behavior [5,6,7,8], husbandry [9,10], disease [11,12,13,14], genetics [15,16], and reproductive physiology [17,18,19,20,21,22,23,24,25,26,27,28,29]. Despite our expanding knowledge base on cheetahs, the species still pose significant reproductive challenges when under human care that does not appear to be shared by free-ranging populations. For almost the last decade, only about 20% of the SSP population has been reproductively successful and as many as 28% of adults are unavailable for breeding each year due to health, behavior or participation in education/ambassador programs [2]. This relatively high proportion of reproductively excluded individuals has been the driving force for research efforts to advance assisted reproductive technologies (ART) for this species and increase the proportion of the population, contributing offspring to SSP population sustainability efforts.

While significant progress has been made in ART procedures related to sperm cryopreservation [22,30,31], exogenous hormone administration to control ovarian activity [32,33], and successful in vitro fertilization, embryo development and transfer [28,34,35], there remains a paucity of information surrounding physiological events occurring post-ovulation in the cheetah. Consequently, the successes of ART, particularly artificial insemination (AI) procedures in this species, are low; only around 22% carried, mostly by a high proportion of early successes in the 1990s that has not been able to be replicated [21,33]. During these procedures, out of 74 females treated with various tested exogenous hormone stimulation protocols, only 49% or 66% of these responded as intended with fresh corpora lutea observed at the time of AI [21,33]. Without a full understanding of the natural physiology and hormonal control of the ovarian cycle and uterine environment through ovulation and successful pregnancy, efforts cannot be made to adjust exogenous hormone stimulation protocols for AI to mimic or encourage these conditions more accurately for this species to contribute to increased pregnancy success.

As nonseasonal induced ovulators, the physical act of mating in cheetahs results in the release of luteinizing hormone (LH) and ultimately, ovulation [29,36]. Although details of uterine events and associated timelines following mating and ovulation in the cheetah are largely unknown, fecal monitoring of progestagen metabolite concentrations and extrapolation from studies performed with domestic cats (*Felis catus*) has provided some information. Similar to the domestic cat [37], in cheetahs, there appears to be no signal from the developing embryo necessary for maternal recognition of pregnancy, with elevated progesterone output maintained throughout gestation (~93 days; [36]) or roughly two-thirds of the pregnancy length if fertilization is not successful, i.e., a non-pregnant luteal phase (NPLP). Therefore, fecal progestagen metabolite concentrations are indistinguishable between pregnant and non-pregnant cheetahs for 55 days or more after breeding or exogenous hormone stimulation and AI [24,36]. Within the cheetah SSP population, lack of offspring production and apparent NPLP occurs in as many as 50% of bred females, even following multiple copulations with the same male [2]. Recent studies suggest that a lowered reproductive potential may be due, at least in part, to external factors related to the captive environment and management of this species in ex situ facilities [7,10].

Much of what is currently known about reproductive biology, health and welfare in zoological facilities in cheetahs has been learned through studies measuring concentrations and patterns of fecal steroid hormone metabolites; many of which were performed in our laboratory [5,7,9,10,24,25,26,27,33,36,38,39,40,41]. While the relationship between the hypothalamic-pituitary-adrenal and the hypothalamic-pituitary-gonadal axes has been long-established to be complex and varies between species [42], prior to this study, estrogen and glucocorticoid concentrations had not been fully described in cheetahs after natural breeding or AI. A recent study in ocelots, which compared steroid hormones in successful pregnancies between naturally bred and embryo transfer events, found changes in estrogens, progestagens and glucocorticoids in embryo transfer pregnancies, which may be responsible for the typically low birth rates with assisted reproductive technologies in felids [43]. In general, and often over-simplistically referred to as “stress” hormones, glucocorticoids are responsible for the response and recovery from energetic perturbations to an organism’s homeostasis and are therefore also associated with metabolism and routine metabolic functions, such as digestion, sleep-wake cycles, and importantly, reproduction [44]. Without a clear understanding of the patterns and relationships between ovarian and adrenal hormones during successful pregnancies, investigations into potential reasons for reproductive failure cannot progress. A previous study in cheetahs revealed a positive correlation between estrogens and glucocorticoids that differs in females between anestrual and cyclic ovarian periods [39], but up to this point, no comparisons in glucocorticoids had ever been made between females that successfully give birth and those that exhibit an NPLP after ovulation.

The current study utilized opportunistic fecal sampling from females in the SSP that occurred over a 9-year period, which had been primarily subjected to hormone analysis to assist with pregnancy diagnosis. Our objective was to examine and compare fecal progestagen (fPM), estrogen (fEM), and glucocorticoid (fGM) metabolite concentrations post-ovulation in pregnant and non-pregnant animals to better understand female physiology in three primary areas where information has been lacking: (1) during successful pregnancies, (2) surrounding frequent non-pregnant luteal phases, and (3) after AI to improve the low success rate. As evidence of recent difficulties obtaining AI success, none of the AI events captured for this study resulted in the production of offspring. All successful pregnancies mentioned hereafter were the result of natural breeding events. As a secondary objective, we also aimed to compare fecal hormone metabolite concentrations during confirmed cheetah pregnancies according to known pregnancy characteristics, parity and litter size.

To address our objectives, fecal samples were collected while females were in one of three ovulatory conditions, one being the pregnant condition, confirmed by the birth of cubs. The other two ovulatory conditions were both when the females experienced an NPLP, but these events were split into two groups based on if the NPLP was following natural breeding, categorized by detection of a luteal response following observed breeding (referred to as ‘NPLP natural’), or exogenous hormone stimulation and AI, but no cubs resulted (called ‘NPLP AI’).

In addition, monitoring fPM in females after breeding remains the cheapest, most efficient, and least invasive method of pregnancy detection within the SSP population, requiring no specialized equipment or animal training [2], but frequent fecal sample collection and shipping to a laboratory is inconvenient for some facilities breeding cheetahs. Fecal samples also require extensive processing before steroid hormone analysis can occur. Here, as a supplemental aim, we also sought to validate a urinary progestagen detection assay for cheetahs and demonstrate utility in pregnancy detection that would require the collection of only one or two samples with no processing needed prior to analysis on our enzyme immunoassay.

## 2. Materials and Methods

### 2.1. Animals and Husbandry

The study was conducted in strict accordance with recommendations in the Guide for the Care and Use of Laboratory Animals of the National Institutes of Health. All samples required for this project were available in our biorepository and were collected non-invasively (feces or urine), therefore not requiring specific IACUC approval or special permits. A total of 13 female cheetahs (*n* = 12 feces sampled; *n* = 6 urine sampled; Table 1) were included in the study; housed at five institutions throughout the USA, all of which were accredited by the Association of Zoos & Aquariums. Study animals were adults of appropriate breeding age (range: 3.6 to 11.3 years of age) at the time of sample collection.

Samples were collected opportunistically over a nine-year period from females that were scheduled for natural breeding (on the basis of SSP breeding management recommendations) or to be given exogenous gonadotropins and subsequent semen deposition via artificial insemination. Specific details regarding exogenous hormone stimulation protocols using equine chorionic gonadotropin (eCG) and then human chorionic gonadotropin (hCG) or porcine luteinizing hormone (LH) are found elsewhere, along with comprehensive procedural details for semen collection, handling, and artificial insemination [21,32,45]. Pregnancy was verified by the birth of offspring. An NPLP was confirmed by an increase in fecal progestagen metabolite concentration after natural breeding or gonadotropin (eCG/hCG or eCG/LH) administration, but no offspring were produced. Some individual females were sampled multiple times through subsequent ovulation events.

### 2.2. Sample Collection and Preparation

Feces were collected for a minimum of 3×/week for at least 2 weeks prior to breeding/exogenous hormone injection—designated the ‘PRE’ time period. Samples continued to be collected through 92 days post-breeding/exogenous hormone injection and were broken into trimesters (as described below) for comparison to the PRE period. Each continuous fecal collection series on a female constituted a single ovulation event. Approximately 50 g of deposited fecal samples were collected <24 h after deposition into individual plastic bags and stored at −20 °C until shipping to the laboratory at the Smithsonian Conservation Biology Institute for analysis. All fecal samples were lyophilized and pulverized, steroid hormone metabolites were extracted, and steroid extraction efficiency was evaluated as previously described [46,47]. The overall mean (±SEM) extraction efficiency for all samples was 77.5% ± 0.2%. Fecal extracts were diluted 1:20 to 1:10,000 in BSA-free phosphate buffer (0.039 M NaH_2_PO_4_, 0.061 M Na_2_HPO_4_, 0.15 M NaCl, H_2_O; pH, 7.0) for analysis by enzyme immunoassay (EIA). Fecal hormone data were expressed as μg/g dry feces.

Urine samples were collected a minimum of 1×/week from the day of breeding or hCG/LH injection through to 91 days after, when possible. Samples of ~3–5 mL were collected immediately after deposition by aspiration from a nonporous surface and stored at −20 °C until analysis. Baseline values were determined by opportunistically collecting samples (*n* = 42) from four of the six females utilized for urinary assessment during time periods determined to be non-ovulatory based on fPM analysis. The urinary progestagen metabolite concentrations for these samples were averaged to create a baseline value for comparison to ovulatory event values. Raw samples were diluted 1:10 to 1:800 in BSA-free phosphate buffer for analysis by enzyme immunoassay (EIA).

### 2.3. Fecal Glucocorticoid Metabolite (fGM) Analysis

Glucocorticoid metabolite concentrations in diluted fecal extracts were determined using a cortisol EIA validated previously in our laboratory for the cheetah [7,38]. The polyclonal antibody used (R4866; C. Munro) was raised in rabbits against cortisol-3-carboxymethyloxime linked to bovine serum albumin. The antibody cross-reacted with cortisol (100%), prednisolone (9.9%), prednisone (6.3%), cortisone (5%), and <1% with corticosterone, desoxycorticosterone, 21-desoxycortisone, testosterone, androstenedione, androsterone, and 11-desoxycortisol. EIA procedures were consistent with what has been reported previously by these authors in this laboratory for analysis of cheetah fecal samples [39]. Samples that were considered too dilute (binding > 80% of maximum) were evaluated at 1:10, and those that were too concentrated (binding < 20%) were assessed at 1:200. The sensitivity of the assay at a maximum binding was 3.9 pg/well.

The inter-assay coefficients of variation (CV) for two internal controls were 10.3% (mean binding, 29.2%) and 6.5% (mean binding, 67.8%), and the CV for all duplicate samples was maintained at <10% (*n* = 65 assays). Serially diluted, pooled fecal extracts expressed displacement curves parallel to the cortisol standard curve (y = 0.889x + 7.169, R^2^ = 0.97, *p* < 0.001). Recovery of added cortisol standard to a fecal extract demonstrated significant recovery (y = 0.9x − 18.0, R^2^ = 0.99; *p* < 0.05).

### 2.4. Fecal Estrogen Metabolite (fEM) Analysis

Estrogen metabolite concentrations in diluted fecal extracts were determined using an estradiol EIA validated for use in the cheetah [19,39]. This EIA relied on a polyclonal anti-estradiol antibody (R4972; C. Munro, University of California, Davis, CA, USA) that cross-reacted with 17β-estradiol (100%), estrone (3.33%), and <0.01% with estrone sulfate, progesterone, testosterone, cortisol and corticosterone. Microtiter plates were assessed as above for glucocorticoid metabolite analysis and as previously described in this laboratory by these authors [39]. Samples that were considered too dilute (binding > 80% of maximum) were assessed at a higher concentration (1:10), whereas those that were too concentrated (binding < 20%) were run at a lower concentration (1:200). The sensitivity of the estradiol EIA at a maximum binding was 1.95 pg/well.

The inter-assay CV for two internal controls were 10.4% (mean binding, 27.3%) and 16.7% (mean binding, 69.4%), and CV for all duplicate samples was maintained at <10% (*n* = 84 assays). Serially diluted, pooled fecal extracts expressed displacement curves parallel to those of the estradiol standard curve (y = 1.104x − 1.473, R^2^ = 0.99, *p* < 0.001). Recovery of added estradiol to a fecal extract demonstrated significant recovery (y = 1.3x − 4.0, R^2^ = 0.99; *p* < 0.05).

### 2.5. Fecal and Urinary Progestagen Metabolite Analysis

Progestagen metabolite concentrations in diluted fecal extracts were determined using a monoclonal antibody assay routinely applied in our laboratory (no. CL425, Quidel Co., San Diego, CA, USA) [19,24,33]. This antibody cross-reacts with 4pregnen-3,20-dione (100%), 4-pregnen-3α-ol-20-one (188%), 4-pregnen-3β-ol-20-one (172%), 4-pregnen11α-ol-3,20-dione (147%), 5α-pregnan-3β-ol-20-one (94%), 5α-pregnan-3α-ol,20-one (64%), 5α-pregnan-3,20-dione (55%), 5β-pregnan-3β-ol-20-one (12.5%), 5β-pregnan-3,20-dione (8%), 4-pregnen-11β-ol-3,20-dione (2.7%), and 5β-pregnan-3α-ol-20-one (2.5%). Microplates were prepared as above for the estradiol assays, and as previously described by the authors in this same laboratory [24].The inter-assay CV for two internal controls was 11.7% (mean binding, 31.9%) and 17.7% (mean binding, 73.5%), and the CV for all sample duplicates was <10% (*n* = 78 assays). The intra-assay CV of two controls at multiple points across a microplate was <10%. Serial dilutions of fecal extract yielded a displacement curve parallel to the standard curve (y = 1.14x − 21.25, R^2^ = 0.895, *p* < 0.001). There was no evidence of matrix interference as adding diluted fecal extract to standards did not alter the amount observed (y = 1.17x − 3.15, R^2^ = 0.985, *p* < 0.001).

Urinary progestagen metabolite concentrations were determined using the same assay as described above for feces, except for 3 of the 14 ovulation events (1 in the pregnant and 2 in NPLP natural conditions), which were analyzed using a different EIA methodology (described below) with the same monoclonal antibody (CL425). In this method, a double antibody system was used, including a secondary goat-anti mouse IgG antibody (A008; Arbor Assays, Ann Arbor, MI, USA). Microtiter plates were first exposed to secondary antibody (0.15 mL; 10 μg/mL), which was given time to attach to the plastic well surface before the second period of incubation with blocking solution (0.25 mL; X109, Arbor Assays). Plates were allowed to dry at room temperature and stored with a desiccant at 4 °C until use. Standards (0.05 mL) were added to pre-coated 96-well microplates in triplicate, and diluted; unknown urine samples (0.05 mL) were added in duplicate. At this time, a horseradish peroxidase enzyme-conjugated hormone (0.050 mL; 1:110,000; C. Munro, University of California) and specific primary antibody (0.050 mL; 1:50,000) were also each added to each well as quickly as possible, and the plate and its contents were allowed to incubate at room temperature for 2 h. Unbound components were removed using a washing procedure for fecal analysis, and a chromogen solution (0.1 mL, Moss, Inc., Pasadena, MD, USA) was then added to each well. Optical densities were determined using a microplate reader (Dynex MRX, reading filter at 450 nm, reference filter at 540 nm).

Serially diluted standard curves produced by this double antibody EIA method were parallel and comparable to the original method, utilizing the same primary antibody; therefore, the reported internal control variation and analytical validations are combined for these two methods with cheetah urine samples. The inter-assay CV for two internal controls was 14.1% (mean binding, 32.4%) and 18.5% (mean binding, 71.8%), and the CV for all sample duplicates was <10% (*n* = 31 assays). The intra-assay CV of two internal controls at multiple points across a microplate was <10%. Serial dilutions of fecal extract yielded a displacement curve parallel to the standard curve (y = 0.867x + 0.73, R^2^ = 0.955, *p* < 0.001). There was no evidence of matrix interference as adding diluted fecal extract to synthetic standards did not alter the amount observed (y = 0.942x − 0.141, R^2^ = 0.999, *p* < 0.001). Normalization of hormone concentrations for variations in urine water content was done by quantitatively measuring urinary creatinine (CRT) and dividing the sample hormone values by CRT concentration, as has been described previously [48,49].

### 2.6. Statistical Analysis, Calculations and Definitions

Ovulation event lengths were measured from the natural breeding date to the parturition date for pregnant females. For NPLP females, the ovulation event length was calculated from the natural breeding or hCG/LH administration date to the date when progestagen metabolites dropped to baseline concentrations. The trimester length was determined by dividing the mean pregnancy length for all females by three. For urinary progestagen metabolite analysis, females in both NPLP conditions, NPLP natural and NPLP AI, were combined to create a single NPLP condition for comparison to pregnant females. Individual urine sample concentrations were averaged by week post-breeding or hCG/LH injection and then combined into three-week terms of weeks 1–3 (term 1), weeks 4–6 (term 2), and weeks 7–9 (term 3) for statistical comparison between pregnant and NPLP females.

Hormone metabolite data were summarized using an overall mean of all samples for the identified condition or trimester, and a baseline of all values was calculated using an iterative process, excluding values greater than the overall mean plus 1.5 standard deviations [36,38,39]. Peak frequency was calculated by dividing the total number of peak samples (see below definitions) by the total number of samples collected for that ovulation event (expressed as a percent) [39]. Mean peak amplitude was determined as the mean height of hormonal peaks above individual baseline values [39]. A significant increase in progestagen metabolite concentration was declared when the levels were three times baseline for ≥five consecutive days following breeding or hCG/LH injection [33]. An estrogen peak was the highest value within a group of samples > 1.5× baseline [36,39]. Values greater than three times baseline were considered to be glucocorticoid peaks [38,39].

Differences in fecal hormone metabolite concentrations among the three ovulatory conditions were determined using a linear mixed-effect model in R package ‘nlme’ (R version 4.0.2) with a gamma distribution that included the condition and term as fixed effects with the individual, housing facility and age at sampling, each as random effects. A separate model with gamma distribution was used to compare trimesters within pregnant females only, as well as ovulatory events that resulted in singletons (*n* = 5) versus multi-cub litters (*n* = 10), and those confirmed pregnant ovulations that produced first-time cubs for a female, primiparous, compared to those of subsequent pregnancies for each female, multiparous. This model held term, cub number and parity categories as fixed effects, and the individual and their age at sampling as random effects. Tukey’s test was performed to determine individual post hoc comparisons between significant groups. Unless otherwise noted, the predicted values were used to generate values for figures. The differences in urinary progestagen metabolite concentrations between pregnant and NPLP females, by term, were also determined using a linear mixed model, including the condition and the interaction between condition and term as fixed effects, and the ovulatory event number nested inside individual as a random effect. Values are reported as mean ± standard error of the mean (SEM) and effects were considered significant at *p* < 0.05. Raw fecal and urinary data can be found in Tables S1 and S2, respectively.

## 3. Results

Descriptive fecal hormone metabolite characteristics for each ovulatory condition, trimester and values from studies using comparable methodologies are found in Table 2. The observed high mean fEM peak amplitude for pregnant females during the PRE time period was driven by two high outlier values, one each from two females housed at the same institution, and did not correspond with significantly high pregnant fEM for this time period within the statistical model.

### 3.1. Ovulatory Condition and Time Period Comparisons

**Figure 1:**
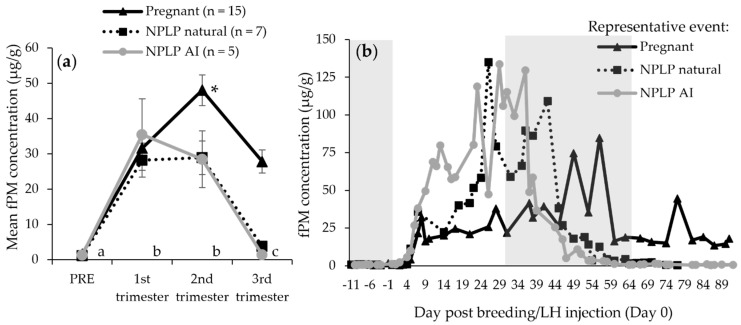
Time period raw means (**a**) and three representatives (**b**) fecal progestagen metabolite (fPM) profiles for female cheetahs experiencing ovulatory events: pregnant, confirmed by birth of cubs, or a non-pregnant luteal phase (NPLP) following natural breeding or exogenous hormone stimulation and artificial insemination (AI), but no cubs resulted. In (**a**), different lower-case letters denote differences (*p* < 0.05) among time periods for all ovulatory events. Asterisk indicates a difference from other trimesters in the pregnant condition only. In (**b**), alternatively shaded sections indicate PRE time period and three trimester divisions.

Overall fPM concentrations for the three ovulatory conditions followed a predictable pattern in time periods following breeding or LH/hCG injection (hereafter abbreviated to LH injection) for females in all conditions, with all trimesters higher than PRE concentrations (*p* < 0.0001; Figure 1). Third trimester concentrations for all ovulatory events were lower than the 1st and 2nd trimesters (*p* < 0.0001), but the first two trimesters were not different from each other (*p* = 0.392). Across all samples, pregnant condition values were higher than both NPLP natural (*p* = 0.0433) and NPLP AI (*p* = 0.0414) conditions.

**Figure 2:**
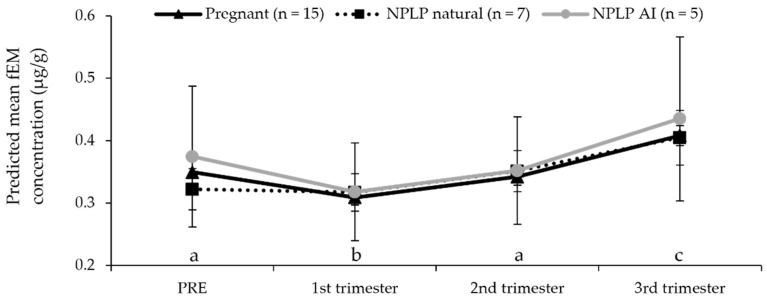
Time period predicted mean concentrations for fecal estrogen metabolites (fEM) from female cheetahs experiencing ovulatory events: pregnant, confirmed by birth of cubs, or a non-pregnant luteal phase (NPLP) following natural breeding or exogenous hormone stimulation and artificial insemination (AI), but no cubs resulted. Different lower-case letters denote differences (*p* < 0.05) among time periods for all ovulatory events.

**Figure 3:**
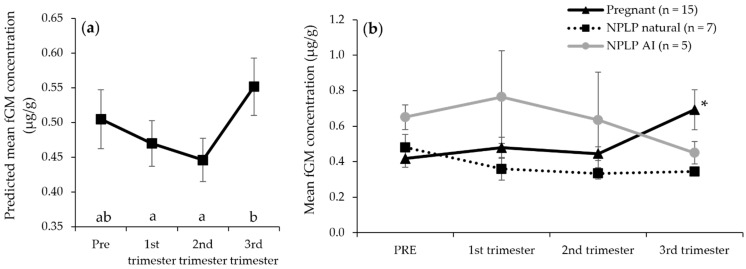
Predicted (**a**) and raw (**b**) fecal glucocorticoid metabolite (fGM) time period means for female cheetahs experiencing ovulatory events: pregnant, confirmed by birth of cubs, or a non-pregnant luteal phase (NPLP) following natural breeding or exogenous hormone stimulation and artificial insemination (AI), but no cubs resulted. In (**a**), all ovulatory events were combined to determine overall model-predicted means. Different lower-case letters denote differences (*p* < 0.05) among time periods. Asterisk indicates a difference (*p* < 0.05) from all other time periods in the pregnant condition only.

Although neither NPLP condition was different from overall pregnant fEM concentrations (*p* > 0.05), the NPLP conditions did differ from each other with NPLP AI fEM being higher (*p* = 0.0459) than NPLP natural fEM concentrations (Figure 2). When all ovulatory conditions were combined, fEM concentrations were lower in the 1st trimester (*p* < 0.001) and higher in the 3rd trimester (*p* < 0.001) than the pre-breeding/LH injection time period (PRE). There was no difference among any ovulatory condition in fGM concentrations (*p* > 0.05), or between any trimester and the PRE time period (*p* > 0.05). However, the 1st and 2nd trimester were both lower in fGM concentrations (1st: *p* = 0.006; 2nd: *p* < 0.001) than the 3rd trimester (Figure 3a).

### 3.2. Fecal Hormone Metabolite Concentrations in Confirmed Pregnant Females

The mixed model, including only pregnant female hormone values, determined all trimester fPM concentrations to be higher (*p* < 0.0001) than PRE concentrations, and the 2nd trimester was higher still than the 1st or 3rd trimester (*p* < 0.0001), which were not different from each other (*p* = 0.174; Figure 1a). In the pregnant condition, fGM were higher in the 3rd trimester than PRE and both earlier trimesters (*p* < 0.0001; Figure 3b). For pregnant females only, fEM concentrations mirror the results of when all ovulatory conditions were combined and were lower in the 1st trimester (*p* < 0.001) and higher in the 3rd trimester (*p* < 0.001), than the pre-breeding/LH injection time period (PRE; Figure 2).

**Figure 4:**
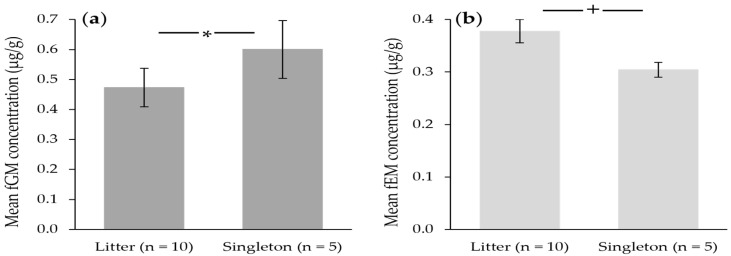
Raw (**a**) fecal glucocorticoid metabolite (fGM) and (**b**) fecal estrogen metabolite (fEM) means for pregnant female cheetahs that gave birth to a litter of more than one offspring (*n* = 10) compared to hormone concentrations collected during pregnancies in which females gave birth to a singleton cub (*n* = 5). Asterisk denotes significance (*p* < 0.05), and plus sign denotes a tendency (*p* = 0.063).

In primiparous ovulatory events (raw mean = 0.405 ± 0.074 µg/g), fGM were lower (*p* = 0.009) than for multiparous pregnancy events (raw mean = 0.595 ± 0.066 µg/g). Interestingly, fGM were also lower (*p* = 0.021) and fEM tended to be higher (*p* = 0.063) during pregnancies of litters of more than one cub, than pregnancies where females only gave birth to a singleton (Figure 4). There was no difference (*p* > 0.05) in fPM concentrations by parity or the number of cubs born in each pregnancy event.

### 3.3. Urinary Progestagen Metabolite Analysis

**Figure 5:**
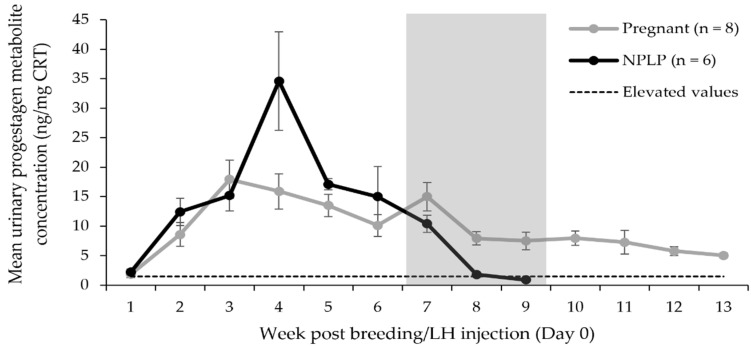
Weekly raw mean urinary progestagen metabolite concentrations in samples collected from female cheetahs after natural breeding or hormone administration and either giving birth to offspring (pregnant), or exhibiting fecal progestagen metabolite concentrations indicative of ovulation, but no offspring were produced (NPLP). Elevated values were determined to be all those above a value three times a non-ovulatory mean baseline urinary progestagen metabolite concentration (1.5 ng/mg CRT). The shaded area indicates the three-week term in which pregnant values were higher than NPLP concentrations (*p* < 0.001).

Mean urinary progestagen metabolite concentrations for each week post-breeding/LH injection are represented in Figure 5. Urinary progestagen metabolite concentrations in pregnant females were higher (*p* = 0.0006) in term 3, representing values from weeks 7–9 combined, than in females experiencing NPLP. The high value in week 4 for the NPLP condition was due to two irregularly high values from one female collected during that period. The statistical model, in controlling for individuals, did not determine a difference between ovulatory conditions for the term including these high values. The considerable discrepancy in all measured concentrations between pregnant (mean: 7.5 ± 1.5 ng/mg CRT; range: 3.1–18.5 ng/mg CRT) and NPLP (mean: 0.9 ± 0.1 ng/mg CRT; range: 0.5–1.6 ng/mg CRT) conditions were observed by week 9, allowing the pregnancy diagnosis to be performed with 100% accuracy for these samples (*n* = 28) using a 2.0 ng/mg CRT cutoff for pregnant values at day 56 post-breeding/LH injection.

## 4. Discussion

Despite an extensive history of scientific investigation, the cheetah remains a species with significant gaps in our understanding of basic and especially reproductive biology. Breeding in the wild is rarely observed, and efforts to achieve a self-sustaining ex situ population have thus far been unsuccessful [2]. While some of the causes for this are likely related to aspects of the captive environment and suboptimal husbandry, there are also unique reproductive challenges posed by this species in human care that dramatically reduce the reproductive potential of the population [7,10]. For example, females exhibit seemingly random periods of anestrus that have, to date, not been attributed to any specific environmental or physiological parameter [33,36,39]. Instances of apparent non-pregnant luteal phases after even multiple copulations with proven males occur in the North American Species Survival Plan (SSP) population with surprising frequency [24]. Successful hormonal stimulation and pregnancy outcomes from artificial insemination (AI) attempts remain at a low frequency in this species, despite much previous research [21,33]. To expand our knowledge base of the physiological events that occur post-ovulation in the cheetah, to improve AI success and uncover areas of focus for future research on this species, we utilized a sample set, almost a decade in the making, of fecal material collected from female cheetahs and sent to the laboratory at the Smithsonian Conservation Biology Institute for assistance with pregnancy diagnosis. Using these samples, that were collected surrounding ovulatory events, such as natural breeding or exogenous hormonal administration for AI, we were able to not only describe estrogen and glucocorticoid concentrations and patterns for the first time in this species after ovulation but also to compare ovarian and adrenal hormone metabolite concentrations between ovulatory events that result in cubs and those that do not. Significantly, we validated a urinary progestagen metabolite assay and generated pregnant and non-pregnant female profiles to assist with the ease of pregnancy detection in this species, requiring minimal sample collections and processing.

Pre- and post-ovulatory fPM concentrations reported here mirror those determined by previous studies on cheetahs using the same assay procedures [25,36,40], depicting an increase from negligible concentrations—occurring at a mean of 5–6 days after natural breeding or LH/hCG administration—to peak concentrations of approximately 120 µg/g that occurred early in the second trimester for NPLP females, and later in this same trimester for pregnant females (Table 2). The slightly (1–2 day) extended time period determined here for initial fPM elevation to occur, from previously published values, is likely due to differences in sampling technique. Some females in the current study were sampled three times per week, possibly missing days of initial fPM increase, and therefore, contributed to an artificial extension in this period, determined from studies in which females were sampled daily [40]. Overall, pregnant females had higher fPM concentrations than both NPLP groups, with the difference evident in individual profiles by the end of the second trimester, or between days 55 to 60 post-breeding/LH injection, again reflecting what has been found previously in this species [24,36]. Although third trimester fPM concentrations were found to be higher in both NPLP groups than samples from the PRE time period, this may be because some of these values were still in the process of returning to PRE levels for some females, causing enough difference to be significant in very low values. Additionally, fEM concentrations were higher for all ovulatory conditions in the third trimester than PRE, indicating NPLP females during this time were likely experiencing typical developing follicular waves and were capable of ovulating. The pregnancy length, from breeding to the birth of cubs, was found here to be 92.6 ± 0.4 days, which was also within published ranges for cheetahs. Although, to our knowledge, the current study includes the largest number of pregnancies (*n* = 15) ever assessed together for this species.

Similarly, pre-ovulatory fEM values that have been previously published for this species were not perceptibly different from those determined in this study (Table 2). Measuring fEM concentrations post-breeding/LH injection showed a decrease in concentrations from PRE values exhibited during the first, followed by an increase in the third trimester. Although they are often compared, domestic cats exhibit a sharp decline and continued low values in circulating estradiol after breeding, associated sometimes with an increase immediately prior to parturition [50]. In contrast, female cheetahs in this study showed similar estrogenic activity after ovulation to the pre-breeding/LH injection values (Table 2). Regular fEM peaks, similar in amplitude to the pre-ovulatory peaks and likely corresponding with waves of follicular development—although dampened somewhat in the first trimester—were detectable in females throughout pregnant and NPLP events. This pattern of fEM concentrations has been noted to occur in other non-domestic felid species, such as the snow leopard and the lion [51,52], as well as domestic mares and heifers during pregnancy [53,54]. In mares, this tendency has even made it possible to expand the genetic contribution of high-quality females through the collection of oocytes during pregnancy for IVF and embryo transfer to a surrogate. During the AI procedures assessed for this study, we observed higher overall fEM for NPLP AI compared to NPLP natural ovulatory events, however, this result appears to be driven primarily by differences in pre-breeding/LH injection concentrations. However, as NPLP AI fEM concentrations were expectedly affected by exogenous hormone stimulation (eCG) at levels higher than what may be produced naturally, and neither NPLP condition was different from the pregnant condition (Figure 2), this difference is, therefore, unlikely to be biologically relevant.

By analyzing this extensive dataset from cryo-repository samples, we were able to investigate fGM concentrations and patterns for the first time in female cheetahs after breeding/LH injection. Our analysis did not reveal any difference in fGM among ovulatory events that resulted in pregnancy and those that exhibited an NPLP, but a more focused analysis of fGM during pregnancy did yield some interesting differences. Expectedly, fGM were higher in the third trimester of pregnancy than any other time period, a pattern exhibited by many other mammals, as higher concentrations are necessary for the final stages of development for various fetal organs, and reflect an increased metabolic demand on the female, ultimately helping to initiate parturition [55,56]. Somewhat unexpectedly, though, primiparous pregnancies were also discovered to produce lower overall fGM concentrations than multiparous pregnancy events. This result is contrary to findings in other species that gestation is more energetically taxing for first-time mothers [57,58], or that there is no impact of parity on measured glucocorticoid concentrations [59,60,61]. A recent publication in wild geladas suggested that because first birth typically occurs prior to the female achieving adult body mass, pregnancy tends to be more energetically taxing because the dam is also supporting her own continued development, thus contributing to higher glucocorticoids measured during primiparous pregnancies [62]. This is not the case for female cheetahs in the SSP, however, as the youngest individual to give birth in this study was 3.6 years in age, and females in this population are known to achieve adult body mass by 1.75 years and are fully through puberty by 2.5 years [27]. Instead, the relationship between parity and measured glucocorticoids may be species-specific. In cheetahs, in the absence of other contributing factors, fGM may be higher due to their anti-inflammatory actions, which are needed during pregnancy to prevent the rejection of the embryo(s) by the mother’s body, requiring a higher response to overcome subsequent, similar exposure that occurs in later pregnancies [56].

Another interesting result in fGM concentrations was the higher overall fGM during pregnancies that resulted in singletons compared to those producing litters of more than one offspring. Although parental investment and the provisioning of resources to multi-cub litters would seem to equate to a higher energetic demand and therefore a higher production of glucocorticoids to meet these demands in females gestating more than one fetus, a similar result was also recently found in domestic cats that gave birth to large vs. small litters [61]. These authors concluded that glucocorticoids and potentially the overall metabolism may have been higher in females leading up to, as well as during gestation of small litters, only allowing for a lower investment in reproduction, and therefore supporting the production of fewer offspring than those females with lower metabolic demands prior to and during gestation [61]. Our results here agree with this hypothesis, aided further by our observed tendency for fEM to be lower in pregnancies, resulting in singletons rather than those of more than one cub. Following this hypothesis, higher glucocorticoids in the period preceding ovulation could result in fewer or smaller developing oocytes, thus lower estrogen production and fewer resulting embryos. Our results are also supported by studies in rodents, finding that a higher total cortisol during gestation was associated with smaller litters, in which the authors suggest a glucocorticoid-mediated mechanism inducing embryonic mortality [63,64,65].

During our investigation of fecal hormone metabolite concentrations after ovulation, we also sought to validate a urinary progestagen metabolite assay to assist with pregnancy determination in cheetahs; one that removes the need for frequent, multi-month sampling efforts. Although urine collection requires a nonporous surface for the aspiration of deposited urine, we were able to develop and compare full pregnant and NPLP urinary progestagen metabolite profiles using the same antibody and EIA procedures our laboratory uses to analyze fPM in cheetahs. Our results have made it possible to successfully diagnose pregnancy from a single urine sample collected on day 56 or later following breeding or LH injection in this species. This outcome decreases resource expenditure needed for institutions breeding cheetahs to diagnose pregnancy, avoiding the need for specialized equipment or extensive fecal collection, storage, shipping, and processing. Resources instead may be put toward the minimal training required to facilitate reliable urine collection from females housed predominantly on porous material.

## 5. Conclusions

For the first time, by utilizing a sample set collected opportunistically over a nine-year period from females around the SSP recommended breeding time or artificial insemination, for analysis for pregnancy diagnosis, we were able to fully describe and compare ovarian and adrenal hormone output after ovulation in pregnant and NPLP cheetahs. Some of the major results we experienced include the discovery of regular fEM fluctuations, likely corresponding to the developing follicular waves occurring after ovulation. These were seemingly not impacted by a luteal presence and/or a developing pregnancy. While fGM were not different among pregnant and NPLP ovulatory events, confirmed pregnant animals did show an anticipated increase in fGM during the third trimester. Although this seems to eliminate fGM involvement in the high frequency of NPLP after successful breeding in this population, our findings of fGM output differences related to parity and litter size seem to indicate a strong role of glucocorticoids in pregnancy of the cheetah that we have yet to fully elucidate. It may be that adrenal output immediately before and during pregnancy could be reducing litter size in this species, although an additional study in this area is warranted. Here, we were also able to validate and generate full profiles of urinary progestagen metabolites after natural breeding and AI, providing a novel technique for pregnancy detection that requires minimal resource and sample collection investment for cheetah breeding facilities. In conclusion, this study has served to fill significant remaining gaps in the physiological events surrounding ovulation and gestation in the cheetah and provided critical information to focus future work in this area.

## Figures and Tables

**Table 1:** The number of female cheetahs and number of distinct ovulation events sampled by feces and or urine collection in each of the following conditions, pregnant or experiencing a non-pregnant luteal phase following natural breeding (NPLP natural) or exogenous hormone stimulation and AI (NPLP AI), but no cubs resulted.

Condition	Females	Ovulation Events
Pregnant	Feces: 8; Urine: 4	Feces: 15; Urine: 8
NPLP natural	Feces: 5; Urine: 2	Feces: 7; Urine: 4
NPLP AI	Feces: 4; Urine: 1	Feces: 5; Urine: 2

**Table 2:** Ovarian and adrenal fecal hormone metabolite characteristics of cheetahs that ovulated and gave birth (pregnant), ovulated after natural breeding and did not give birth (natural non-pregnant luteal phase; NPLP natural), ovulated after exogenous hormone administration for artificial insemination, and did not give birth (AI non-pregnant luteal phase; NPLP AI). Ovulation events were measured from breeding or hCG/LH administration (abbreviated as LH injection) date to birth for pregnant females and date progestagen metabolites dropped to baseline concentrations for NPLP females. Values shown are group means ± SEM.

Parameter	Pregnant (*n* = 15)	NPLP Natural (*n* = 7)	NPLP AI (*n* = 5)	Published Value ^1^ [Source]
**Fecal progestagen metabolites:**				
Time to elevation (days)	5.9 ± 0.48	5.57 ± 0.43	5.8 ± 1.28	3–4 [40]
Length of ovulation event (days)	92.60 ± 0.40	60.29 ± 4.75	55.20 ± 2.73	Preg: 94.2 ± 0.5 [36] Preg: 92.8 ± 0.4 [40]
Pre-breeding/LH injection baseline (μg/g)	1.58 ± 0.38	1.10 ± 0.36	1.28 ± 0.74	0.92 ± 0.2 [25]
Mean of elevated values (μg/g)	38.32 ± 3.58	30.74 ± 4.63	36.99 ± 9.22	40–70 [40] Preg: 28.7 ± 6.5 [25] NPLP: 43.9 ± 12.7 [25]
Peak (μg/g)	120.78 ± 18.86	97.14 ± 16.57	110.12 ± 30.82	
Peak post-breeding/LH injection (day)	39.60 ± 4.33	31.14 ± 2.52	29.8 ± 4.73	
First trimester mean (μg/g)	31.63 ± 3.61	28.12 ± 4.80	35.43 ± 10.13	
Second trimester mean (μg/g)	48.05 ± 4.37	28.91 ± 4.79	28.45 ± 8.02	
Third trimester mean (μg/g)	27.85 ± 3.30	3.775 ± 1.37	1.43 ± 0.47	
**Fecal estrogen metabolites:**				
*Pre*-breeding/LH injection–				
Mean (μg/g)	0.34 ± 0.03	0.31 ± 0.04	0.41 ± 0.16	0.26 ± 0.02 [33] 0.29 ± 0.01 [39] 0.33 ± 0.07 [25]
Baseline (μg/g)	0.26 ± 0.02	0.26 ± 0.05	0.35 ± 0.14	0.21 ± 0.02 [33] 0.22 ± 0.01 [39]
Peak frequency (%)	10.06 ± 2.22	14.08 ± 2.00	14.75 ± 3.40	
Mean peak amplitude (μg/g)	0.75 ± 0.35 ^2^	0.22 ± 0.04	0.27 ± 0.16	
*Post*-breeding/LH injection–				
Mean (μg/g)	0.36 ± 0.02	0.36 ± 0.04	0.32 ± 0.06	Preg: 0.32 ± 0.06 [25] NPLP: 0.28 ± 0.05 [25]
Baseline (μg/g)	0.24 ± 0.01	0.28 ± 0.04	0.20 ± 0.02	
Peak frequency (%)	21.53 ± 2.03	15.00 ± 1.43	17.01 ± 2.28	
Mean peak amplitude (μg/g)	0.34 ± 0.03	0.29 ± 0.04	0.33 ± 0.10	
First trimester mean (μg/g)	0.29 ± 0.02	0.33 ± 0.04	0.36 ± 0.07	
Second trimester mean (μg/g)	0.37 ± 0.02	0.35 ± 0.04	0.29 ± 0.06	
Third trimester mean (μg/g)	0.44 ± 0.05	0.45 ± 0.05	0.32 ± 0.06	
**Fecal glucocorticoid metabolites:**				
*Pre*-breeding/LH injection–				
Mean (μg/g)	0.42 ± 0.05	0.48 ± 0.07	0.65 ± 0.07	0.98 ± 0.13 [33] 0.62 ± 0.07 [39]
Baseline (μg/g)	0.25 ± 0.04	0.29 ± 0.02	0.40 ± 0.05	0.60 ± 0.12 [33] 0.32 ± 0.04 [39]
Peak frequency (%)	12.25 ± 5.02	8.22 ± 3.78	14.10 ± 4.67	
Mean peak amplitude (μg/g)	0.47 ± 0.20	0.84 ± 0.40	1.63 ± 0.15	
*Post*-breeding/LH injection–				
Mean (μg/g)	0.53 ± 0.06	0.35 ± 0.05	0.61 ± 0.17	
Baseline (μg/g)	0.28 ± 0.03	0.26 ± 0.04	0.43 ± 0.19	
Peak frequency (%)	14.42 ± 2.99	4.60 ± 2.06	15.44 ± 4.19	
Mean peak amplitude (μg/g)	1.25 ± 0.20	0.48 ± 0.18	1.82 ± 0.71	
First trimester mean (μg/g)	0.48 ± 0.06	0.36 ± 0.06	0.77 ± 0.26	
Second trimester mean (μg/g)	0.45 ± 0.04	0.33 ± 0.03	0.64 ± 0.27	
Third trimester mean (μg/g)	0.69 ± 0.11	0.34 ± 0.02	0.45 ± 0.06	

^1^ Previously published value(s) from studies utilizing the same or similar hormone analysis methodology. ^2^ Noted high values driven by outlier values from two females at the same location.

## Data Availability

The data presented in this study are available in Supplementary Material Tables S1 and S2.

## Supplementary Materials

The following are available online at https://www.mdpi.com/article/10.3390/ani12070809/s1, Table S1: raw fecal hormone data, Table S2: raw urinary hormone data.
